# Feasibility and Tolerance of Fingertip Peripheral Arterial Tonometry Measurements in School-Aged Children

**DOI:** 10.3389/fped.2021.622056

**Published:** 2021-05-05

**Authors:** Karolien Van De Maele, Roland Devlieger, Steven Provyn, Jean De Schepper, Inge Gies

**Affiliations:** ^1^Division of Paediatric Endocrinology, Department of Paediatrics, University Hospital Brussels, Brussels, Belgium; ^2^Research Unit Organ Systems, Department of Development and Regeneration, Catholic University of Leuven, Leuven, Belgium; ^3^Research Unit Research Group Growth and Development (GRON), Vrije Universiteit Brussel, Brussels, Belgium; ^4^Department of Obstetrics and Gynaecology, University Hospital of Leuven, Leuven, Belgium; ^5^Anatomical Research and Clinical Studies, Vrije Universiteit Brussel (VUB), Brussels, Belgium

**Keywords:** clinical research, peripheral arterial tonometry, procedural pain and distress, children, non-therapeutic research

## Abstract

**Background:** Assessment of the endothelial function of the microvasculature by peripheral arterial tonometry (PAT) has gained increasing popularity in pediatrics. Discomfort or experienced pain during fingertip PAT has only been studied in adolescents and adults.

**Methods:** In 142 children (aged 4–11 years old), a fingertip PAT with a commercial device (EndoPAT 2000®) as well as a caliper and ultrasound examination of peripheral skinfolds were performed as part of a cross-sectional cohort study. In 110 children, Faces Pain Scale (FPS-R) data were collected after PAT and skinfold measurements by caliper and ultrasound.

**Results:** In 111 out of the 142 PAT measurements (78.2%), a reactive hyperemia index (RHI) could be obtained. The most frequent error messages by the software was a “too noisy” and/or a “poor quality” signal. The success rate was higher in children aged older than 6 years (83.1 vs. 44.4%; *p* < 0.001). Median (range) FPS-R after PAT was 0 (range 0–6) but was significantly higher than the median pain experienced after caliper measurements of peripheral skinfolds (*p* < 0.001). No pain was experienced by 59 of the 110 children (54.1%).

**Conclusion:** PAT testing is feasible in the great majority of school-aged children, and the procedure is well-tolerated.

## Introduction

Peripheral arterial tonometry (PAT) measurement enables researchers and clinicians to assess endothelial function in a non-invasive manner, in arteriosclerosis risk populations, such as familial hypercholesterolemia, type 1 diabetes, and severe obesity (1–9). By fingertip PAT, the hyperemic reaction after an arterial occlusion of the upper arm of 5 min, called the Reactive Hyperemia Index (RHI), is measured with a thimble-shaped finger cap that imparts a systematic pressure at the distal phalanx of the index fingers (10–12).

Researchers face several challenges performing these measurements in children (13). The first main challenge is to provide adequate and child age-appropriate reassurance before starting the test. This is to try to diminish the fear of the finger clamping and the eventual pain of discomfort experienced by the upper arm compression, as anger and anxiety are associated with lower RHI (14). While pain has been reported by a small percentage, and the discomfort associated with the arterial occlusion was rather described as “a tingling feeling” or “nearly painless” by either adolescent or adult patients, the tolerance of PAT testing in school-aged children has not been reported (13, 15).

A second challenge is to obtain reliable measurements with the device, originally developed for use in adults, which poses in children certain potential disadvantages, as meticulously described by Bruyndonckx et al. leading to a higher risk of error messages generated by the inbuilt software (13, 16). Surprisingly, most pediatric PAT studies do not report on the amount of encountered error messages.

Therefore, we analyzed the tolerance and usefulness of the fingertip PAT in school-aged children. We expect a good pain tolerance of the technique, given the previous experience in adolescents, but a lower success rate in obtaining reliable software readings, especially in the younger children and in those children with a more somatizing or anxiety behavioral type.

## Methods and Materials

### General Study Information

This study is performed as part of the EFFECTOR-study (17). The EFFECTOR-study was designed as a cross-sectional cohort/assistant study, investigating the long-term vascular and metabolic outcome of the offspring of different maternal cohort studies, comprising a total of 143 children.

**Figure 1:**
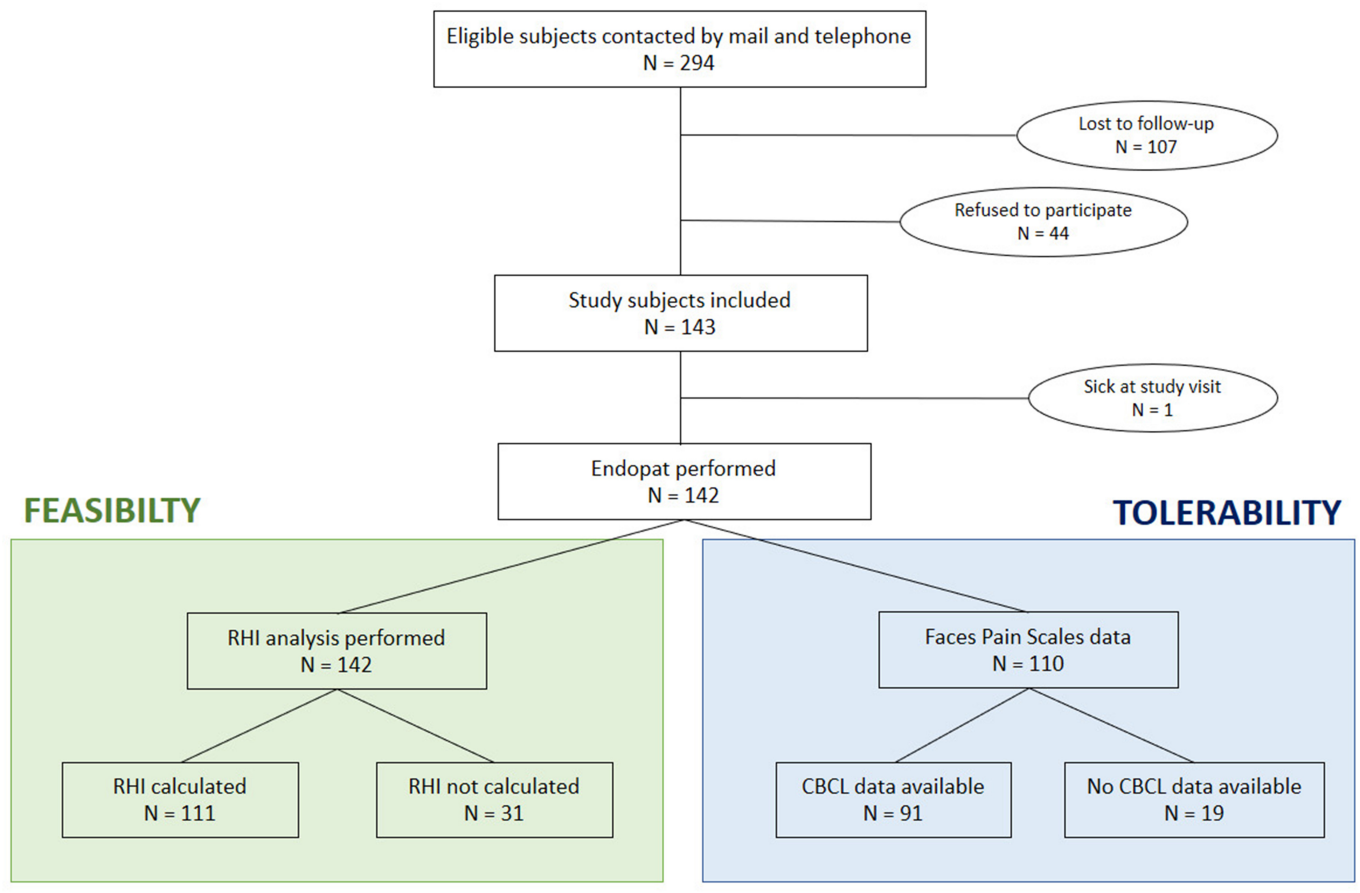
Flow chart of patient inclusion. RHI, reactive hyperemia index; CBCL, child behavior checklist.

In Figure 1 an overview of the inclusion flow is given. All eligible study subjects did receive a letter by mail. One to two weeks later, they received a text message. Afterward, there were at least two attempts to contact them by phone call and at least one message was left on their voicemail. When the phone number was no longer in use, a second letter was sent by mail.

In 142 children an EndoPAT measurement was performed. One child was excluded because of a fever at the time of the study (Figure 1). In addition, peripheral body fat assessments, using calipers, and ultrasound measurements were performed in all children (17).

The study is registered at ClinicalTrials.gov with the unique number NCT02992106 and is approved by the Ethics Committees of the UZ Brussels and UZ Leuven/KU Leuven. In an amendment to the study, the analysis of the pain experienced by the children during the PAT and the skinfold measurements was approved. Written informed consent was obtained from the parents, and each child received age-adjusted information through an assent.

### Studied Outcomes

According to the study protocol (17), data on endothelial function and peripheral fat accumulation were collected during a single home visit. All visits were performed by the same trained pediatric fellow, KVDM.

The EndoPAT 2000® device from Itamar Medical Ltd was used for the PAT analysis. PAT measurements were performed according to the standard operating procedure for measurements in children as meticulously described by Bruyndonckx et al. (13). For this measurement, both index fingers of the patient are placed in pneumatic probes. First, the device performs 5 min of baseline measurement of pulse wave amplitudes. Then, a blood pressure cuff is inflated to a suprasystolic pressure (average pressure + 10–20 mmHg) to occlude the arterial flow of the non-dominant arm for 5 min. After a rapid deflation of the cuff, a reactive hyperemia takes place, which is a measure for the arterial endothelial function. The software package calculates the RHI. A higher RHI value represents a better endothelial function. Observations on behavior and complaints during the test were noted.

The Harpenden skinfold caliper (Baty International Ltd, RH15 9LB England), putting a constant spring pressure of 10 g/mm^2^ on the skinfolds, was used according to the ISAK guidelines (18). Measurements were performed at four sites (biceps, triceps, subscapular, and supraspinal). At the same sites, an ultrasound measurement of the fat layer was performed with a linear 7.5 Mhz probe (Mindray Diagnostic Ultrasound System, model M7, Mindray®, Bio-Medical Electronics co., LTD, Shenzhen, China) using gentle pressure.

Children were asked to fill out a Faces Pain Scale (FPS-R) immediately after the PAT as well as the skinfold measurements by caliper and ultrasound. Since this was added as an amendment to the study after start of data collection process, data are available only from 110 children (Figure 1). In the children aged older than 6 years, the Child Behavior Checklist (CBCL) was filled in by the parents to screen for eventual psychopathology (19). The somatizing and anxiety scores at the CBCL were calculated and analyzed to correlate with the data from the FPS-R.

### Statistical Analysis

Statistical Analyses were performed using the SPSS IBM version 25 software package. Since the reported FPS-R scores had a skewed distribution, non-parametric statistical analyses were used throughout the analysis. Friedmans test of differences or the Wilcoxon Signed Ranks test was used for comparisons. Correlations were analyzed by the Spearman rank correlation coefficient. *P-*values below 0.05 were considered statistically significant.

## Results

### Population Characteristics

The median age of the 142 studied children was 10.5 years (range 4.2–13.2 years). There were 70 boys (49.3%) and 72 girls (50.7%). Median (range) weight standard deviation (SD) score, height SD score, and body mass index (BMI) SD score were, respectively, 0.2 (−2.0–3.0); 0.3 (−1.3–3.3); and −0.1 (−3.1–2.5). There was a prevalence of 16.2% of children with overweight (BMI SD score of 1.3–2.3) and of 2.8% of children with obesity (BMI SD score >2.3). Median (range) systolic blood pressure SD score and diastolic blood pressure SD score were, respectively, 0.3 (−1.7–2.8) and 0.1 (−1.7–1.6) (20, 21).

The children who had a FPS-R analysis, showed comparable characteristics as the whole studied population. They had a maximum age of 11.4 years (data not shown). The CBCL scores, available from 91 children, ranged from 50 to 78 on the somatizing domain and from 50 to 88 on the anxiety domain. An abnormal anxiety score was observed in eight children (8.9%).

### Feasibility of PAT

**Figure 2:**
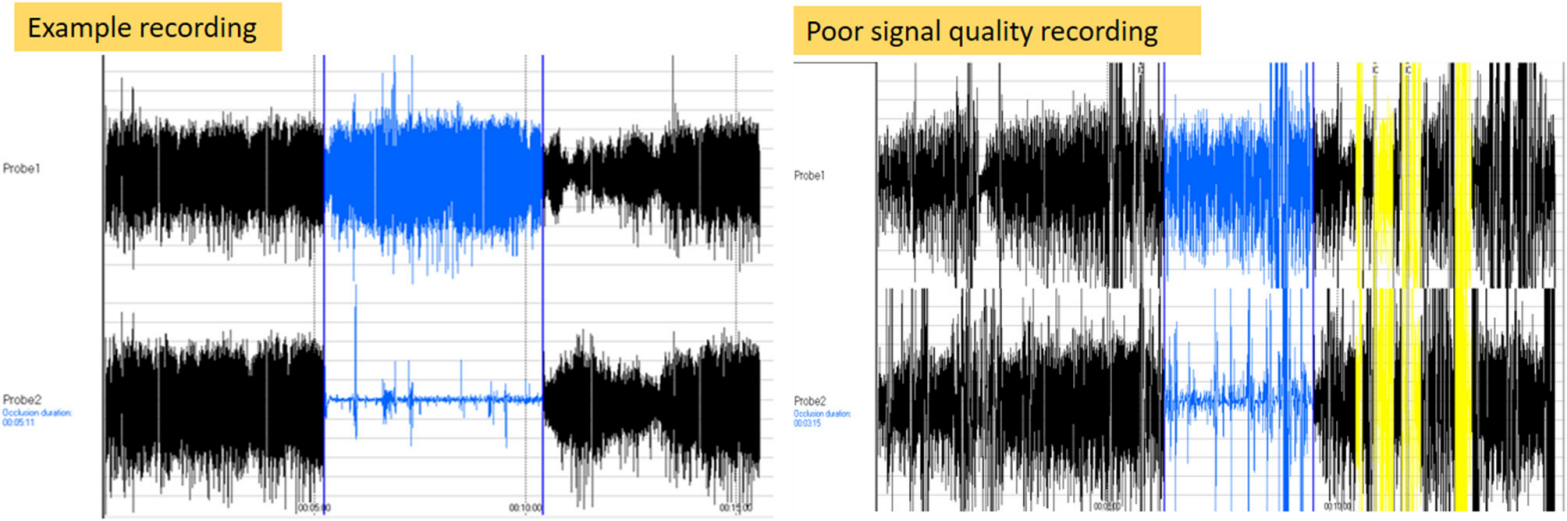
Illustration of error recording. The left side of the figure shows a signal of a recording with a desired pulse wave pattern. The right side of the figure shows a recording with a poor signal recording.

The RHI was generated by the software package in 111 children (78.2%). The most frequent error messages were a signal that was too noisy or that had poor quality. Figure 2 shows an example of a desired PAT recording in comparison to a PAT recording with an error message. Observations made during the failed 31 RHI measurements, as well as other characteristics of these children are summarized in Table 1. A young age and behavioral problems were found to be the major limiting factors to generate a PAT result. However, in 11 of the 31 children, no explanation for the failed RHI was present. In only 8 of the 18 children younger than 6 years, the RHI was generated (44.4%), while results were obtained in 103 of the 124 children older than 6 years (83.1%) (*p* < 0.001). In the group of children older than 6 years, the CBCL anxiety scores did not differ between the groups whether the RHI was obtained (*p* = 0.41).

**Table 1:** Overview of probable explanations of failed RHI calculation.

	**Children without calculated RHI (*n =* 31)**
Young age (below 6 years old)	9/31 (29.0%)
Children diagnosed with a condition that might provide an explanation	
Autism spectrum disorder	2/31 (6.5%)
ADHD	1/31 (3.2%)
Gilles de La Tourette	1/31 (3.2%)
Known behavioral problems	1/31 (3.2%)
Could not keep fingers still (no specific behavioral problem)	5/31 (16.1%)
Vasovagal reaction	1/31 (3.2%)
No obvious explanation	11/31 (35.5%)

### Tolerability of PAT

#### Comparison of the FPS-R Results Between the Technical Procedures

**Figure 3:**
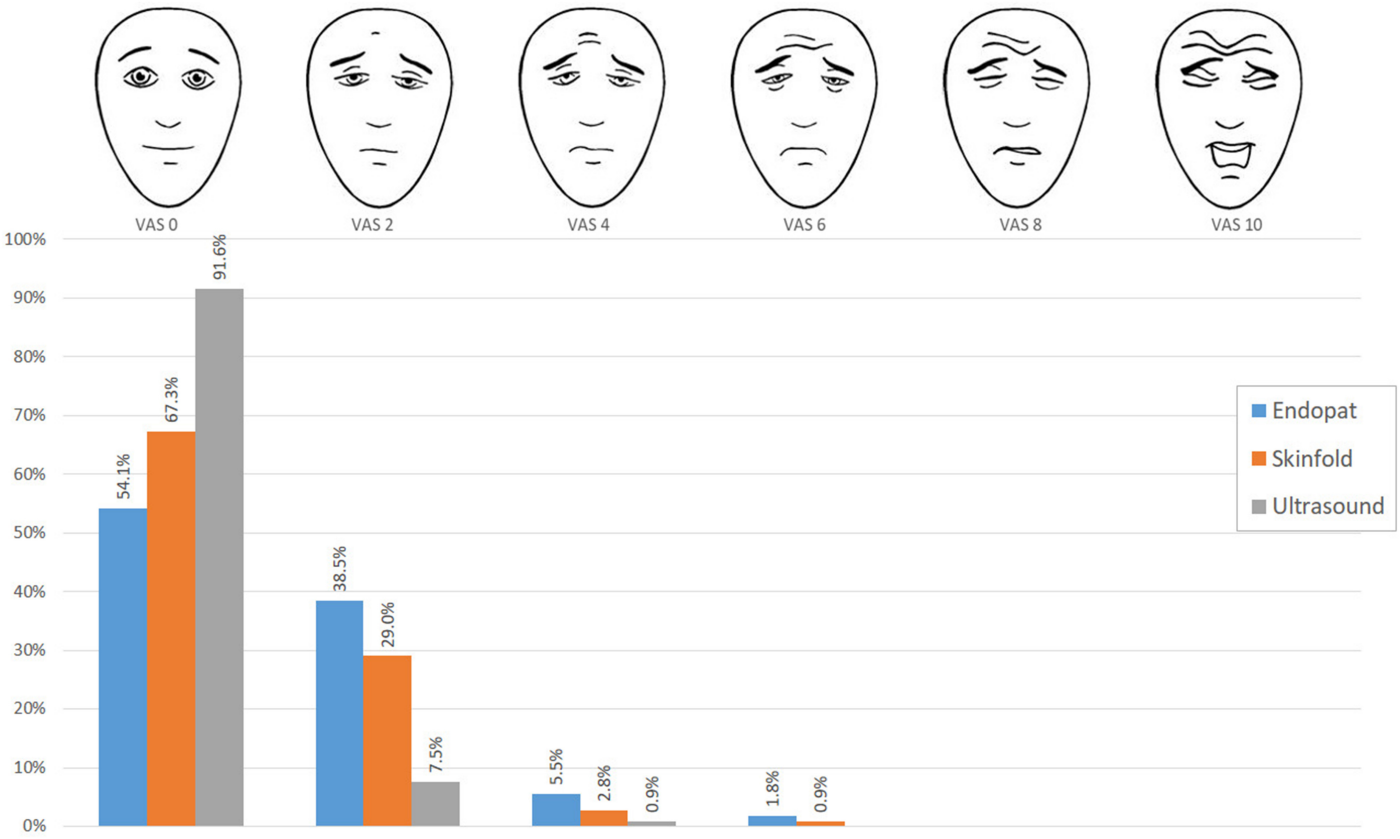
Distribution of FPS-R scores. The blue bars indicate the FPS-R scores filled out after the PAT measurement, the orange bars indicate the FPS-R scores after skinfold measurement, and the gray bars show after the ultrasound measurement.

The FPS-R results of the PAT, caliper assessment, and ultrasound are presented in Figure 3. FPS-R 0 was the median pain score, after all three measurements. The maximum values were FPS-R 6 after PAT and caliper measurements and FPS-R 4 after the ultrasound examination. The proportion scoring 0 (no pain) was the lowest after PAT measurements (54.1%) and highest after the ultrasound measurements (91.6%). The median FPS-R was highest after PAT (*p* < 0.001). Pairwise *post-hoc* testing confirmed the highest median FPS-R score after the PAT, followed by the median FPS-R score after the caliper measurement and lowest median VAS score after ultrasound measurement.

#### Correlates of the FPS-R Scores After PAT

No gender differences in the FPS-R score were found (*p* = 0.98). No significant correlations between the FPS-R score and pain score after the caliper examination (R = −0.05; *p* = 0.58) or ultrasound examination (R = 0.03; *p* = 0.76) were present. The FPS-R scores were not significantly correlated with the age of the children (R = 0.15; *p* = 0.12), the duration of the arterial occlusion (R = 0.02; *p* = 0.84), or the somatizing or the anxiety score of the CBCL (respectively, R = −0.13; *p* = 0.23 and R = 0.002; *p* = 0.99).

## Discussion

We have examined for the first time the tolerability of fingertip PAT in a cohort of young children. We found that the experienced pain in school-aged children is tolerable, however more intense than the discomfort experienced during skinfold measurements by caliper. Recordings were of sufficient quality in three-quarters of the examinations, unrelated to gender or a more anxious behavioral type.

We used the FPS-R as an instrument for pain assessment, because it is validated to assess pain from the age of 3 years up to adolescence and is the recommended pain scale to use in the research context (22). A previous study in 30 healthy adolescents (aged 13–20 years) showed a median pain score of 1 out of 5 using a Visual Analog Scale (23). The median score in our study was 0. A difference of one face is in general considered the smallest clinically significant difference (24). Of importance, the PAT examination is found to be painless in by more than half of the children, who were examined in a home situation.

It is well-known that reported procedural pain consists of both modifiable and non-modifiable determinants, such as age and gender (25). Our data do not support a decrease in pain intensity with increasing age. The CBCL questionnaire provided information on certain personality traits. We did not found that female subjects or subjects having a more anxiety type of behavior, as assessed by the CBCL questionnaire, were at higher risk (25).

Regarding the modifiable determinants, several interventions to alleviate the fear of the examined children were taken. First, measurements were made in a home situation. Additionally, all children received age-appropriate information through a visually attractive assent and video material (26). During the measurement, they were offered distraction by watching a television program (26). These interventions are simple and easily applicable. However, since we do not know to what extent certain variables in the home environment might have influenced the measurements, a future control group with measurements performed in a standard hospital environment might offer additional insights (27).

Since we experienced a drastic drop in our success rate for the calculation of RHI when children's age was below 6 years (success rate of only 44.4%), we would recommend considering this age as the minimum age to perform the PAT testing in young children. The noisiness of the signal is decisive for the calculation of the RHI by the software package. Therefore, we advise outweighing the need for the test and the expected discomfort for the child in cases of known diagnosis with a behavioral problem. However, each case should be viewed separately.

We can report pioneering data in a large group of school-aged children who underwent testing by the EndoPAT 2000® device in the context of a non-therapeutic study. The FPR-S was used to collect information on the experienced discomfort in a standardized manner. Our study has however several limitations. The performance of the PAT during home visits not controlling for room temperature might have influenced the signal quality. Also, the CBCL questionnaires might not provide us with correct information specific to the pain experience.

Future research should focus on a further detailed differentiation of the pain experience and a more detailed study of the factors interfering with the feasibility and tolerability of the PAT measurements.

In conclusion, our results show that fingertip PAT testing is feasible in school-aged children, with a preferable age above 6 years old. The reported discomfort is tolerable, however more intense than the discomfort experienced during skin fold measurements by the caliper.

## Data Availability Statement

The raw data supporting the conclusions of this article will be made available by the authors, without undue reservation.

## Ethics Statement

The studies involving human participants were reviewed and approved by the Ethics Committee UZ Brussels and the Ethics Committee UZ Leuven/KU Leuven and was registered at ClinicalTrials.gov (NCT02992106). Written informed consent to participate in this study was provided by the participants' legal guardian/next of kin.

## Author Contributions

KVDM, RD, and IG designed the study and conceptualized the study protocol. SP contributed to data acquisition and performed by KVDM. KVDM drafted the initial blueprint, which was reviewed extensively for content and methods by all other contributing authors. All authors approved the final version of the manuscript as submitted for publication.

## Conflict of Interest

The authors declare that the research was conducted in the absence of any commercial or financial relationships that could be construed as a potential conflict of interest.
